# Challenges to Homeopathy in View of the Framing of Modern Medicine: Towards a Proper Understanding of ‘Homeo-patho-logy’

**DOI:** 10.1055/a-2762-4038

**Published:** 2026-02-25

**Authors:** Josef M. Schmidt

**Affiliations:** Institute of Ethics, History, and Theory of Medicine, Ludwig Maximilian's UniversityMunichGermany

**Keywords:** framing, medicine, homeopathy, critical theory, philosophy

## Abstract

The scientific and public discourse on medicine has in the last century been framed in a way that the progress of high-tech medicine, with its methodological restriction on materialistic aspects of the physical body, may appear as a plain success story, from ancient ignorance to modern science—leaving no constructive idea or reason for being of homeopathy. On the other hand, there has always been a tradition of scholarly criticism of medicine, be it historiographic, natural scientific, social or economical. The shortcomings of most conventional forms of critique, however, rest on their embeddedness in the usual one-dimensional thinking: i.e. their unintentional complying with, rather than questioning, the frame of predominant narratives.

A categorial reframing of medicine may be possible on the basis of three recently rediscovered traditions of thinking, termed *lógos-* , *hómoion- and iásthai* -thinking. Within such an enlarged reframing of the scientific and public discourse, not only medical, social, economic, political and cultural problems could be handled and possibly solved on a higher level of consciousness, but also homeopathy, properly understood as ‘homeo-patho-logy’, would benefit and attain a better, much more reasonable standing.

## Introduction

We are living in weird times. On the one hand, theoretically and individually, we appear to be able to look up virtually any information people have ever gathered and documented, thanks to new media such as the internet and artificial intelligence. On the other hand, practically and collectively, it seems that many have become less critical and more ignorant of basic principles of life than any previous generation. What critics have formerly denounced and termed alienation, manipulation, heteronomy, etc., most people today seem to be unaware of, or uninterested in and thus, in fact, are easy victims.

Obviously, this paradox may indicate the success of what sociologists and psychologists call ‘framing’: in other words, an activity, intangible to the common person, to mould and streamline the thinking and communication of citizens, to determine the way data are conveyed, processed and embedded into narratives, conceptions and opinions favourable to the ruling class. In Western countries, for example, people for the most part are convinced that they are living in free, democratic societies that protect and guarantee human rights, such as autonomy, freedom of speech, press and choice, equality and self-determination. Even if, in reality, some may painfully experience the pressure of political correctness, cancel culture, economic extortion, etc., they would not desist from their belief of being free and independent people, because this is what they have learned from childhood.

## Framing of Modern Societies

However, for at least 100 years, it has been recognised and published that democracies are being worked by a kind of ‘invisible government’, out of the reach of election or de-selection by the people. In the 1920s, Edward Bernays 1 and Walter Lippmann 2 3 had described in detail how instruments of manipulation, such as propaganda and public relations, were already being used on a large scale to shape what they called public opinion as well as individuals' market behaviour.

Framing the minds of the masses by a small elite of big players has long since been examined and scientifically proven. Rainer Mausfeld 4 summarises and supplements recent research about how, for instance, violation of moral norms by industrialists, economists, politicians and others can be veiled from the public's awareness by means of fragmentary presentation, de-contextualisation and re-contextualisation.

These techniques of framing are used in democratic regimes as well as in autocracies. Availing themselves of scientific expertise on these methods proves cheaper for governments compared with exerting crude power, bribery or visible suppression. Application of smart techniques such as steering of attention, selective and incomplete reporting, data flooding, induction of fear or apathy, management of indignation or the like, suffices to make their population follow the same line.

The awkward truth about this is that we may all be affected by it. The only difference might be that some may possibly see through it, while the vast majority will fall for it. Framing—that is the intentional construction, dissemination and concurrent concealment of a framework of thinking, perceiving and opining—relates to all fields of social, economic and political interest, including medicine.

## Framing of Modern Medicine

Having this in mind, a critical look may be taken on the general conviction prevailing among most contemporaries that modern medicine has been nothing but an unprecedented success story, from antiquity and the Middle Ages towards an era of enlightenment. According to this narrative, reduced mortality and augmented life expectancy were a direct result of the introduction of chemical pharmaceuticals and vaccinations, technology, molecular biology and so on. Additionally, medical students are convinced of being taught ‘the best medicine ever’.

But where do we ‘know’ this from—or better, on what basis do we assume this? To be honest, how many of us today are in a position to gain experience of life directly through our own senses, unaffected by any sort of cultural, political or scientific framing? Most of us, including doctors, may have learned the story of a powerful, progressing and beneficent high-tech medicine from mass media or from second-hand sources, rather than from our own unbiased contact with reality. As a result of this kind of framing since childhood, public opinion is neatly convinced that technology, statistical evaluation, regulation, standardisation, mass production, among others, are the keys to the advancement of medicine.

The dark side of this widespread modern world view is that complementary methods of medicine, such as homeopathy, would appear to have no place in such a system or framework. It almost necessarily has to be interpreted as being an anomaly, obsolete, fallen out of time, outside the box. Since documented cures cannot be easily ignored, however, they are usually subsumed under the biased label of ‘placebo effects’, and homeopathy is declared as being ‘nothing but placebo’. 5 Hence, homeopaths may have an existential interest in questioning this frame of thinking within which they may not have a fair chance of being understood or accepted, let alone appreciated.

Academically, for more than a hundred years, chairs of history of medicine have eruditely investigated the development of medicine. Since the first chairs were inaugurated by the medical profession at medical faculties, to pool data for substantiating the narrative of a continuous progress, the frame of investigation was from the outset the scheme of advance from dark to light, focusing on technical and pharmaceutical inventions and their heroes. Later histories of medicine, run by social historians rather than by physicians, however, went on to illuminate the socio-economic background and contexts as well, questioning or relativising prejudiced hero-worshipping. 6 Meanwhile, most of the chairs of history of medicine have vanished from universities. They have been substituted by chairs of medical ethics.

## Criticism of Modern Medicine

### Historical Criticism

Yet the narrative of a triumphant and flawless modern medicine with its inviolable methods has been repeatedly contested, by medical historians outside the mainstream. Thomas McKeown, for example, demonstrated in the 1970s that the decrease of mortality in Western countries should not be attributed to medicines or public health measures, but rather to better nutrition and living standards. Especially the decline of most epidemics and infectious diseases, such as tuberculosis, was already underway and almost completed before antibiotics and vaccinations were developed. 7

Harris L. Coulter examined how the founding of the AMA (American Medical Association) in 1847 was a political reaction to the threat caused by the founding of the AIH (American Institute of Homeopathy) 3 years before, in 1844, and how, due to the lack of effective therapies, the so-called regular physicians had to resort to dodgy political means, such as banning consultation with homeopaths or imposing costly institutional standards on homeopathic colleges and hospitals, in order to subsist and finally prevail against their homeopathic competitors. 8

Ted Kaptchuk, in an article in *The Lancet* in 1998, reconstructed how the new Gold Standard of randomised controlled trials (RCTs) was introduced in 1946, at Cornell University, New York, by Harry Gold, against a reluctant medical profession, in order to finally be able to refute ‘the enormous success of homeopathy’ on a sophisticated scientific basis. 9 10

All this critical historical work about recurring campaigns against homeopathy, as important and significant it may be, hardly fits into the predominating framework of a gloriously progressing modern medicine. And that is why these critical accounts may probably have little chance to gain public awareness or impact. In case of incompatibility with the mainstream frame of thinking, isolated facts are always sitting at the shorter end of the lever. As Leon Festinger already noticed in 1957, people dislike ‘cognitive dissonance’. 11

### Scientific Criticism

Apart from criticism by historians of medicine, there also exists, as it were, a natural scientific type of critique. As an example, Ben Goldacre 12 13 revealed and denounced many breaches against the methodology and ethics of evidence-based medicine committed by pharmaceutical companies as well as by individual doctors. Since these frauds might result in distorted evidence and thus in false decisions of medical practitioners, he demands that they should be prevented. As this is his highest horizon of reasoning, Goldacre's stance is entirely embedded in the frame of modern medicine. There is no criticism of its structures, methodology or world view. 14

Peter Gøtzsche 15 16 also documented many cases of malpractice by the pharmacological industry and medical profession, and accused them, on the basis of all data available, of organised crime. As a scientist of statistics, he came to conclusions such as: ‘In the United States and Europe, drugs are the third leading cause of death after heart disease and cancer’, or: ‘Psychiatric drugs kill more than half a million people every year in the West’, or: ‘It would be far better to remove all from the market’.

His striking charges were all substantiated with scientific evidence: that is, deducted from meta-analyses and reviews of RCTs. Clearly, his ultimate horizon (and limitation) of argumentation was evidence-based medicine (EBM), which is itself part of the current framing of modern medicine. The fact that in 2018 Gøtzsche was expelled from the Cochrane Collaboration in Denmark, which he had co-founded 25 years before, may prove that strictly adhering to a set of contemporary scientific standards may not suffice to understand the entanglement of modern medicine and its institutions with industries, foundations and governments, let alone to cope with them in a realistic and pragmatic way.

### Social Criticism

Apparently, these scientific critics of medicine, mentioned so far, seem to be unaware of more profound social criticism performed and documented some 50 years ago, when Ivan Illich was still permitted to publish his theses 17 in *The Lancet* . 18 The focus of his critique was the excessive professionalising and bureaucratisation of modern life, especially the ‘medicalisation’ of society, as this may result in limiting freedom and justice and in corrupting and incapacitating individuals.

Illich introduced the term ‘iatrogenesis’ and claimed that its damaging effects outweigh any positive benefits of modern medicine. On the basis of sociological research, he provided proof for adverse effects of clinical, social and cultural iatrogenesis, the latter paralysing healthy responses of unspoiled people to suffering, impairment and death. His ultimate horizon of affirmation, however—that which he did not question but presuppose—was such values as autonomy, freedom, self-government and democracy, all based on individual voluntarism and thus remaining inside the frame of bourgeois modern thinking.

Still more challenging was the critical theory of the so-called Frankfurt School, as published by Max Horkheimer & Theodor Adorno 19 20 and Herbert Marcuse. 21 They argued that the Enlightenment had failed by giving rise to national socialism, Stalinism, mass consumer capitalism, and other forms of totalitarianism. In advanced industrial societies, by creating false needs, individuals are integrated into a system of production and consumption via mass media, advertising, industrial management, and contemporary modes of thought, resulting in a ‘one-dimensional’ universe of thought and behaviour, in which criticism and opposition are barred from arising at all. Commodification of everything, however, proves to be psychologically, socially and environmentally destructive.

Horkheimer may even have tended to transcend general criticism of the social system, when in his critique of medicine he laid emphasis on particular concrete shortcomings and real experience of pain and suffering, on grounds of the principle that these perceptible realities must always be the starting point of any theory of change. And he may well have had the intuition that rational general critique may only apply to, and thus presupposes, a rationally functioning society. If there were irrational hotbeds of dominance and cruelty within it, a generalising rationality may, as a matter of principle, be unable to grasp them. 22

## The Shortcomings of Conventional Criticism of Medicine

Reviewing the criticism of medicine in the last five decades, it seems that its horizon has shrunken—from a radical and uncompromising challenge of the socio-economic basis of the medical system and frame, as in the Frankfurt School, to cautious conformist denouncements of scientific flaws within a societal system and frame that is no longer really questioned any more, in some bestselling books of an economically framed market.

The common denominator or frame of all criticism of medicine and society, so far considered, is a secular, hedonist and voluntaristic world view with a focus on the basic demands of the masses. But if all the shortcomings and contradictions pointed out in these theories were resolved, would this be tantamount to paradise on earth—or, more pragmatically, would this be feasible at all?

Empirical studies in anthropology have underpinned the old philosophical argument that humans, struggling for wealth, honour or prestige, power and pleasure in an earthly one-dimensional frame, may necessarily remain unsatisfied, because there is no natural end in these directions. 23 To take an example invoked by Arthur Schopenhauer, it is as if a thirsty person keeps drinking salt water.

To really arrive at a point of rest and completeness, or perfect physical, social and mental health and beatitude, it may be necessary to consider additional dimensions beyond the usual frame of political activism and rational regulations. In view of the potential detriments of untamed dynamics of a reductionist system of medicine, it may be legitimate and seminal to ask for holistic completion.

As it turns out, it is neither necessary nor feasible nor advisable to stall profane rationality for good. But there is an urgent need to, as one might say, cushion it, counter or balance or lead it by orientation towards higher ideas or images that are outside of its reach. Rationality must be guided by an instance that guarantees a good lead, but that may at the same time never be fully ‘understood’: i.e. constructed, deconstructed, verified, falsified, proven, disproven and so on by plain reason. Hence, it may not be possible to demonstrate or name it directly in terms of logic, substantiality or objectivity—though it may be paradigmatically embodied and represented in certain concrete human beings or in certain concepts of the art of healing, such as homeopathy.

## Framing of Homeopathy

Much of the future of homeopathy may depend on how people understand it, by what principles it is agreed to be constituted, what its strengths and core competences are, among others. And here, framing plays a crucial role. It is, so to say, all about lighting control: which aspects should be illuminated in what proportion?

If homeopathy uncritically accepts the frame of the current narrative of a flawless progress of modern medicine by means of and restriction to its scientific methodology and standards, such as EBM and nothing else—it might be lost in the long run.

In fact, as revealed by a wide range of literature, this overly glorious image of computing rationality turns out to have some big blind spots—which, clearly, can never be detected from within the system or frame they belong to. Only when viewed against a different background or assessed from within another frame, may they be realised at all.

## A New Frame for Homeopathy

Recently, multidisciplinary research has shown three powerful traditions of thinking that reach back to Greek antiquity and pervade the cultural and medical history of civilisations all over the world. 24 25 Termed *lógos* -, *hómoion* - and *iásthai* -thinking, they constitute a kind of three-dimensional coordinate system and may be perfect candidates for a new framing of homeopathy and medicine.

Within such an all-encompassing frame, homeopathy naturally appears to embrace all three dimensions, while modern high-tech medicine may be attested an amnesia of *hómoion* - and *iásthai* -thinking, fixating almost exclusively on *lógos* -thinking. This is no trivial matter. In fact, this is the overwhelming chance for homeopaths to realise their own strength and identity, by becoming aware, in a paradigmatic sense, of what they are doing.

### Homeo-patho-logy

As a corollary of this new framing, it may be reasonable to have a new take on the term homeopathy, which literally means ‘suffering by similars’. Adjusted to the new frame of conception, encompassing the three dimensions *hómoion* , *iásthai* ( *patheín* ) and *lógos* , it may rightly read ‘homeo-patho-logy’: that is, the doctrine [ *lógos* ] of (curative) suffering [ *patheín, iásthai* ] by similars [ *hómoia* ].

In this light, *lógos* —as detrimental as its isolated and untamed predominance may have repeatedly proven—should not be underestimated but emphasised as an essential constitutive part of the homeopathic art of healing. It is not about combat or competition between the three principles, but about balancing and keeping their right order and proportion.

If homeopathy is comprehended and framed in such a way, with an emphasis on being constituted by these three dimensions (that have to be continuously balanced by the artist of healing), it would appear to embrace more wisdom and healing art than modern high-tech medicine. Considering that *lógos* -thinking originally arose from the spirit of money, which itself has an intrinsic tendency to expand infinitely without regard to the consequences, there seems to be even more need of some kind of counterpoise to contain the potentially devastating effects of unleashed rationality. 26

## Conclusion and Outlook

Using these categories of a three-dimensional dynamic play of principles, to not only suggest a new framing of homeopathy, but also of medicine at large, the shortcomings of the presently dominating system may appear in a brighter light. Within this new framework, we can no longer just blame missing evidence, injustice, bureaucratisation, medicalisation and the like as original causes of all evils. If this were so, their abolition would result in an ideal state of being. But as long as critics and reformers remain fixed solely on this dimension of abstract rationality, this may never happen.

Only when viewed in a three-dimensional frame, however, does the lack of the other dimensions in modern medicine become apparent. It is missing a sense and awareness of (1) the *hómoion* -dimension, i.e. orientation of human beings to, and connection with, ideas (in a Platonic sense) rather than being merely determined by material causes, and of (2) the *iásthai* -dimension, i.e. comprehension of the dynamics of catharsis as a way of beneficial suffering.

Both principles, in order to be re-introduced into common cultural consciousness, would require cultivation: a lively culture shaped by this frame. In modern civilisation, however, there is little emphasis laid upon these other two dimensions that should always go hand in hand with a healthy rationality. On the contrary, a lot seems to be done to impede such a culture of balancing different dimensions. Indeed, not much respect of tradition, solid education and skillful art is promoted today, but rather voluntarism, egalitarianism, legalism, hedonism, consumerism, and the struggle for a career, for money and for power.

Within a three-dimensional frame (like the one pointed out here), the world would likely be a better place. Medicine would be prevented from economy-driven rationalistic excesses, homeopathy would be recognised as an exemplary art of healing, and homeopaths, being aware of their mission, would have a very different, more reasonable standing.

## Highlights

- In the wake of unprecedented advances in digital data processing and artificial intelligence, the possibilities and risks of manipulation and framing of public opinion become a vital issue, affecting also homeopathy.
- The scientific and public discourse on medicine has in the last century been framed in a biased way, in favour of economy-based high-tech medicine and to the disadvantage of homeopathy.
- Most critical (historical, scientific, socio-economical) analyses and deconstructions of this one-sided framing proved to be insufficient, due to their own embeddedness in the biased narrative.
- A categorial reframing of medicine seems to be necessary and possible on the basis of the three dimensions or traditions of thinking: *lógos* -, *hómoion* - and *iásthai* -thinking.
- Within such an enlarged reframing of medicine, homeopathy would be properly understood as ‘homeo-patho-logy’ and would attain a better, much more reasonable standing.
